# Raman Spectroscopic Studies of Dinaphthothienothiophene (DNTT)

**DOI:** 10.3390/ma12040615

**Published:** 2019-02-18

**Authors:** Bishwajeet Singh Bhardwaj, Takeshi Sugiyama, Naoko Namba, Takayuki Umakoshi, Takafumi Uemura, Tsuyoshi Sekitani, Prabhat Verma

**Affiliations:** 1 Department of Applied Physics, Osaka University, Suita, Osaka 565-0871, Japan; bishwajeet@ap.eng.osaka-u.ac.jp (B.S.B.); sugiyama@ap.eng.osaka-u.ac.jp (T.S.); umakoshi@ap.eng.osaka-u.ac.jp (T.U.); 2 The Institute of Scientific and Industrial Research, Osaka University, Mihogaoka, Ibaraki, Osaka 567-0047, Japan; nnanba@sanken.osaka-u.ac.jp (N.N.); uemura-t@sanken.osaka-u.ac.jp (T.U.); sekitani@sanken.osaka-u.ac.jp (T.S.)

**Keywords:** DNTT, transistor, Raman spectroscopy, DFT calculation, orientation

## Abstract

The application of dinaphthothienothiophene (DNTT) molecules, a novel organic semiconductor material, has recently increased due to its high charge carrier mobility and thermal stability. Since the structural properties of DNTT molecules, such as the molecular density distribution and molecular orientations, significantly affect their charge carrier mobility in organic field-effect transistors devices, investigating these properties would be important. Here, we report Raman spectroscopic studies on DNTT in a transistor device, which was further analyzed by the density functional theory. We also show a perspective of this technique for orientation analysis of DNTT molecules within a transistor device.

## 1. Introduction

Organic field-effect transistors (OFETs) are important electronic devices that are often used in digital screens [1], electronic papers [2], plastic circuits [3], and sensors [4] because of their unique properties, such as flexibility, light weight, ease of large area fabrication, and low-cost production. Among many other kinds, OFETs based on acene and heteroacene organic semiconductor materials have been extensively investigated [5,6]. In terms of better performance due to their high charge carrier mobilities, pentacene molecules and their derivatives in acene series have been widely used in organic electronics [7,8]. However, practical applications of pentacene-based devices are limited due to their instability in the atmosphere, as it degrades and forms pentacene-quinone via a photo-oxidation reaction [9,10].

**Figure 1:**
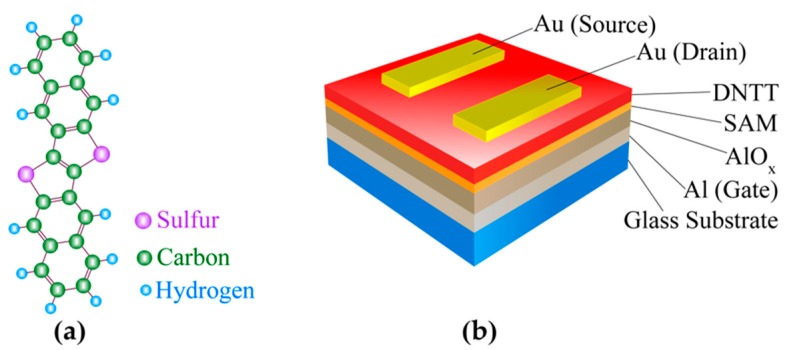
(**a**) Molecular structure of dinaphthothienothiophene (DNTT). Purple, green, and blue circles represent sulfur, carbon, and hydrogen atoms, respectively; (**b**) A schematic of a fabricated DNTT organic field-effect transistor (OFET) device.

Recently, dinaphthothienothiophene (DNTT, C_22_H_12_S_2_), a novel organic semiconducting molecule developed by Takimiya and coworkers, showed improved performance and stability compared to those of pentacene [11]. Since then, a number of publications on the application of DNTT molecules have appeared [12,13,14,15,16]. However, these investigations on DNTT were primarily focused on the practical realizations of the OFET devices and demonstration of their potential applications. On the other hand, the possible effects of the fundamental structural properties of DNTT—such as the molecular density distribution and molecular orientations—on the device performance have not been investigated extensively. The DNTT molecule has a long molecular structure composed of six linearly aligned aromatic rings (Figure 1a) and is known to attach to a substrate in such a way that the long molecular axis remains perpendicular to the substrate [17]. For the best possible charge carrier mobility, the DNTT molecules should be densely packed and aligned on the substrate in such a way that the aromatic rings of all the molecules remain parallel and face each other so that their π-electrons interact with each other, enabling good carrier mobility of the charge carriers. Both low molecular density and a possible molecular twist along its axis are expected to degrade the π-π stacking between molecules, resulting in lower carrier mobility and degraded device performance. Since these structural features affect the electronic properties of transistor devices significantly, it is fundamentally crucial to investigate the molecular density as well as the molecular twists of the DNTT molecules in an actual OFET device for possible improvement in device performance.

There are various techniques to investigate the structural properties of DNTT, such as atomic force microscopy (AFM) and X-ray diffraction spectroscopy (XRD) [18]. Since AFM reveals only topographical properties, it helps in investigating only the morphology of the DNTT film in a device. It is difficult to obtain detailed information about the orientation of the molecules within a morphological domain using AFM. Although XRD can be used to analyze the molecular orientations, the spatial resolution is poor. The orientation information obtained by XRD is spatially averaged out due to the low spatial resolution, and, hence, it is not suitable for investigating the effect of molecular orientations on carrier mobility. In addition, because the XRD technique is based on Bragg reflection, the application of this technique is limited to highly ordered molecules in the DNTT films, and the non-ordered areas cannot be analyzed.

An optical analysis based on Raman spectroscopy would be useful to overcome the above-mentioned limitations [19,20,21]. In this technique, one can analyze the molecular vibrational information of a sample using Raman scattered light generated from the interaction of the light with molecules. A spatially resolved measurement is also possible by combining Raman spectroscopy with optical microscopy to achieve a spatial resolution of a few hundred nanometers. In fact, it is even possible to improve the spatial resolution down to a few nanometers by combining Raman spectroscopy with plasmonic techniques in tip-enhanced Raman spectroscopy [22,23,24,25,26,27]. The molecular density of DNTT can be evaluated from the Raman scattered intensity because the scattered intensity depends on the number of molecules within the focused spot of incident light. Furthermore, some Raman vibrational modes may depend upon the orientation of the molecule with respect to the electric field of the probing light. Therefore, a polarization-dependent Raman investigation can reveal the molecular orientation. In a previous report, we demonstrated that it was possible to analyze the molecular orientation of pentacene, a molecule similar to DNTT, where the tilt angle of the pentacene on the substrate was investigated by using polarization-dependent Raman analysis [28,29]. The DNTT molecules are known to have no tilt as they stand perpendicular to the substrate in the OFET device; however, they may have a twist along their molecular axis. Therefore, in a similar way with the help of polarization-dependent Raman measurements, one can study the twist of DNTT molecules within the focus spot. Further, since it is a non-contact and non-destructive technique performed in ambient conditions, another advantage of Raman spectroscopy is that it can be performed directly on an actual OFET device. Therefore, Raman spectroscopy provides a new aspect to analyze the structural properties of DNTT molecules in the device. However, to the best of our knowledge, there are no reports on the Raman analytical study of DNTT molecules.

In this article, we report Raman spectroscopic studies of DNTT molecules on an actual transistor device to comprehensively understand the fundamental properties of molecular vibrations of DNTT. We found that a typical Raman spectrum of DNTT molecules shows multiple vibrational modes. We investigated the spatial distribution of DNTT molecules through Raman images constructed from the intensity of one of the Raman modes. Further, in order to identify the measured vibrational modes, density functional theory (DFT) calculation was performed using the Gaussian 09 software, which also helped us to describe the nature of the vibrations of the atoms in the observed vibrational modes. Our calculation results indicated that one of the vibrational modes was dominated by the atomic displacements in a particular direction. The intensity of such a vibrational mode can be enhanced if the polarization of the incident light matches this preferred direction of atomic displacement. Therefore, we used the polarization dependence of the intensity of this mode to estimate the orientation of the DNTT molecules. This work contributes to understanding the molecular structural properties of DNTT in electronic devices and should stimulate studies on DNTT-based electronic devices to further improve their electronic performance.

## 2. Materials and Methods

### 2.1. Fabrication of Organic Field-Effect Transistor Device

Figure 1b shows a schematic of a DNTT OFET device. A 30-nm thick aluminum (Al) layer was deposited on a glass substrate (Corning Eagle XG, Santa Paula, CA, USA) using vacuum deposition (ULVAC EX-200, Chigasaki, Kanagawa, Japan), which was then anodized in 1 mM of citric acid to grow an aluminum oxide (AlO_x_) layer. In the next step, a self-assembled monolayer (SAM) of dodecylphosphonate was grown by immersing the substrate in 3 mM of dodecylphosphonic acid (C_12_H_27_O_3_P) for 12 h at 30 °C followed by rinsing in isopropyl alcohol and drying with N_2_ gas. The Al layer serves as the gate electrode and AlO_x_-SAM as the gate dielectric of the transistor. The fabrication process was followed by the vacuum deposition of a 30-nm-thick layer of DNTT molecules that was deposited at a rate of 0.2 Å/s. Finally, a 50-nm-thick gold layer was deposited on the substrate through a shadow mask to fabricate source/drain contacts followed by an annealing of the device at 100 °C for 1 h under vacuum. In the active layer, DNTT molecules stand vertically on the AlO_x_-SAM substrate, which means the long axis of the DNTT molecules is perpendicular to the substrate [17]. This molecular conformation can contribute to a high carrier mobility between the source and the drain of the device due to the π - π stacking between the molecules. After fabrication, we measured the electrical properties of the fabricated OFET device in order to confirm its performance. We found the carrier mobility for this device to be 1.92 cm^2^·V^−1^·s^−1^ at a source-drain voltage of −3 V, which was similar to other reports [15,16].

### 2.2. Raman Measurement

**Figure 2:**
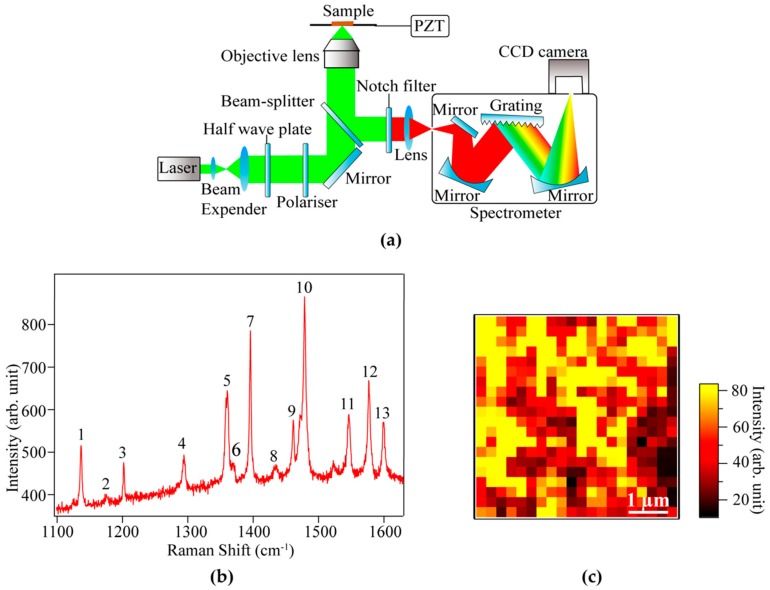
(**a**) A schematic of the optical setup for Raman spectroscopy; (**b**) a typical Raman spectrum of DNTT molecules in our OFET device, measured with an incident laser power of 140 µW and exposure time of 30 s; (**c**) Raman image of the sample constructed by the intensity of peak 10 at 1478.2 cm^−1^.

We measured Raman spectra of the DNTT layer in the OFET device utilizing an optical setup of a backscattering configuration illustrated in Figure 2a. A linearly polarized excitation laser with a wavelength of 532 nm was focused on the sample through a 150× objective lens (NA = 0.95). It should be noted that the absorption wavelength of DNTT molecules is located at 445 nm [30,31]. Since the excitation wavelength of 532 nm is far from the absorption wavelength of the sample, we could avoid the resonant Raman effect in our measurements. Multiple factors may affect Raman intensity near the resonant condition, which makes Raman analyses complicated. The polarization of the laser within the focus spot remained parallel to the substrate as the perpendicular component gets canceled out. The Raman scattered light from the DNTT molecules was collected by the same objective lens, which was then dispersed with a spectrograph (Princeton Instruments, Acton SP2300, Acton, MA, USA) equipped with a grating (1800 grooves/mm), and detected with the help of an EM-CCD camera (Princeton Instruments, Pixis 100, Acton, MA, USA). Rayleigh light was efficiently blocked by a notch filter.

### 2.3. Density Functional Theory Calculation

Density functional theory calculation was carried out by means of the Gaussian 09 package using Becke’s nonlocal three-parameter exchange and the correlated functional with Lee–Yang–Parr correlation (B3LYP) functional method [32,33] and a basis set of 6-311G (d, p).

## 3. Results and Discussion

An example of a typical Raman spectrum measured from the DNTT molecules in an OFET device is shown in Figure 2b, where several vibrational modes can be observed in the spectral range from 1100 to 1650 cm^−1^, which are marked as peaks 1 to 13. We also constructed a Raman intensity image at 1478.2 cm^−1^ (peak 10), shown in Figure 2c, which indicates the density distribution of the DNTT molecules in the device with a spatial resolution of a few hundred nanometers. Higher Raman intensity indicates areas with a larger density of DNTT molecules, while a low density of molecules is expected in the areas with weaker Raman intensity. Peak 10 was selected here to show an example of a Raman image because this peak had the highest intensity. Indeed, Raman images constructed with every peak showed a similar distribution. The Raman intensity image indicates that the density of DNTT molecules was not uniform, which can affect the efficient transport of charge carriers in the DNTT layer of the device. These results suggest that the Raman imaging was significantly helpful for understanding the density distribution of DNTT molecules in the OFET device with a high spatial resolution of a few hundred nanometers, and, hence, can be utilized to improve the fabrication process.

**Figure 3:**
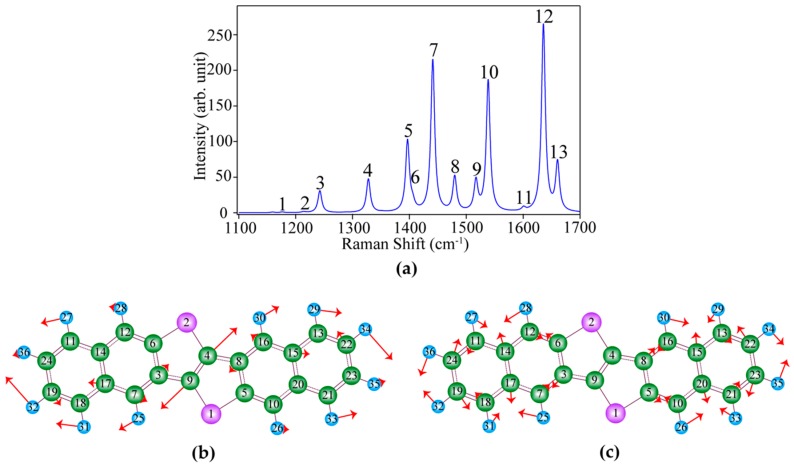
(**a**) A density functional theory (DFT) calculated Raman spectrum of a DNTT molecule; Graphical representations of atomic oscillations for (**b**) peak 10 and (**c**) peak 11.

As mentioned earlier, we performed a DFT calculation to identify the measured vibrational modes in the Raman spectrum of DNTT molecules. An example of the calculated Raman spectrum of a DNTT molecule is shown in Figure 3a. We observed multiple vibrational peaks in the calculated Raman spectrum, which are in good agreement with those found in the experimental spectrum shown in Figure 2b. Although there are slight differences in the two spectra, it is because only a single isolated molecule was considered in the DFT calculation whereas the experiment was performed on a 30-nm-thick film of DNTT molecules on the substrate. By comparing the spectral patterns between the calculation and the experiment, we assigned the numbers 1 to 13 to the various peaks observed in the calculated spectrum.

We further analyzed the nature of each vibrational mode of the DNTT molecule from the results of the DFT calculations, where it was possible to visualize the oscillation strengths and the directions of the displacements of the atoms in a vibrational mode. Figure 3b,c show schematics of the molecular vibrations related to peaks 10 and 11, respectively, as examples, where the directions and the strengths of the atomic displacements are indicated by the directions and the lengths of the red arrows. Here, the length of all the arrows in each vibrational mode was multiplied by a factor of 2.5 from their initial length for clearer visualization. For easy identification, we assigned each atom a number. As an example, in the case of peak 10 in Figure 3b, we observed a strong C–C stretching mode between C(4) and C(9) atoms in aromatic systems combined with a few dominant C–H in-plane bending modes, such as for atoms H(32) and H(34). Although there were a variety of vibrational modes in the Raman spectrum of DNTT molecules, they basically originated from the C–C stretching mode in aromatic systems combined with C–H in-plane bending modes. The representative atomic vibrations of each vibrational mode are summarized in Table 1 and some prominent atomic vibrations in some vibrational modes are discussed in the supplementary information. Furthermore, in the case of the vibrational mode at peak 11, we found that the stretching directions of C(19)–C(24), C(17)–C(14), C(20)–C(15), and C(23)–C(22) atoms are in the direction perpendicular to the long axis of the DNTT molecule, as shown in Figure 3c and in video S5 in the supplementary information. This oscillation characteristic is also similar to the A_g_ mode of pentacene at 1533 cm^−1^, which is polarization dependent, as investigated in a previous report [34]. Although this mode appears relatively weak in the calculated spectrum, it had enough intensity in the experimental spectrum for further analysis of this mode. Therefore, this mode can be used for the orientation analysis of DNTT molecules in experiments through its polarization dependence. If an excitation light polarized in the direction of the vibration of these C–C stretching interacts with the DNTT, the vibrational amplitude and Raman scattering intensity would enhance. In contrast, the vibrational amplitude would reduce when the excitation light is polarized in a perpendicular direction to the C–C stretching.

In order to estimate the twist angles of DNTT molecules through the polarization-dependent intensity of peak 11, we used two mutually-perpendicular linear polarizations of the excitation light, which were parallel to the substrate plane. If we call the direction of the molecular axis as the Z-direction, then the incident light was polarized in mutually perpendicular directions within the X–Y plane. It should be noted that in our previous work [29], we investigated the tilt of the pentacene molecule from its Z-axis toward the X–Y plane. Therefore, we used two mutually perpendicular polarizations, one along the Z-axis and the other in the X–Y plane, so that the ratio of Raman intensities between the two polarizations gave us the value of the tilt angle. Here, in contrast, the DNTT molecules are known to stand along the Z-axis, governed by the fabrication conditions. However, they can twist along the Z-axis resulting in a twist of aromatic rings in the X–Y plane. Therefore, two mutually perpendicular polarizations, both lying in the X–Y plane, would give us the information about molecular twist while they still stand perpendicular to the substrate. If one of the polarizations is along the direction of C–C stretching and the other is in a perpendicular direction, while both lying in the X–Y plane, the Raman intensity would be maximum for the first polarization while it would be minimum for the second polarization. Hence, the Raman intensity ratio for peak 11 at the same position under the two perpendicular polarizations would give information about the twist angle of the standing DNTT molecules. The polarization direction of the excitation light in our experiment was controlled by using a half waveplate and a polarizer in the optical path.

**Figure 4:**
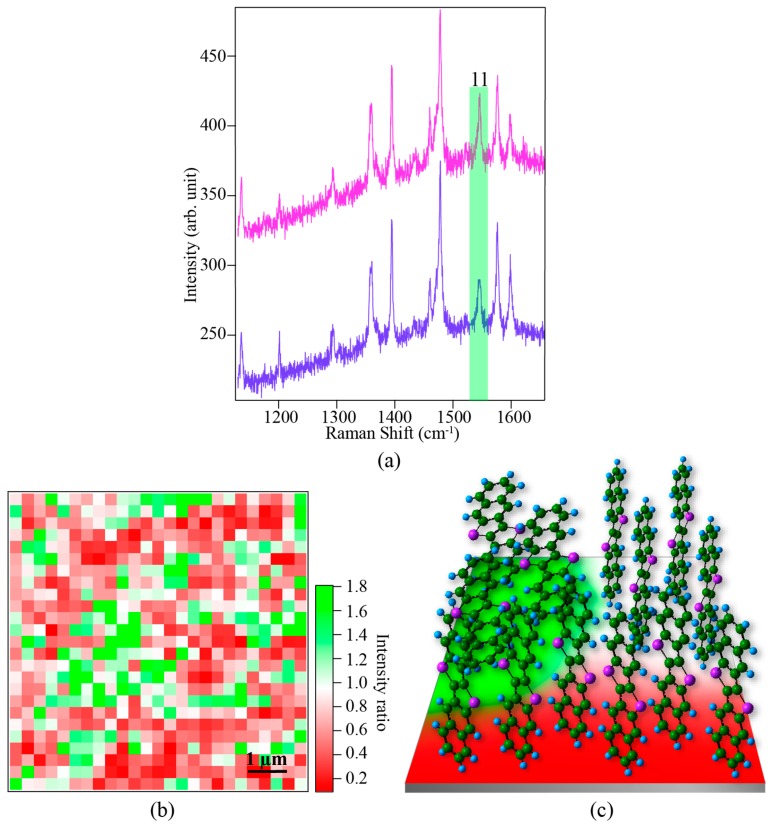
(**a**) Polarization-dependent Raman spectra with two mutually perpendicular linearly polarized light; (**b**) Raman intensity ratio image showing the distribution of molecular twist for the DNTT sample. The image was constructed by dividing Raman intensities of peak 11 obtained from the two mutually perpendicular linearly polarized light; (**c**) An illustration of the orientation of DNTT molecules in the device.

An example of Raman spectra obtained at the same sample position using orthogonally polarized incident light is shown in Figure 4a. Indeed, peak 11 showed a change in its intensity depending on the polarization direction of the incident light, confirming a polarization dependence of this vibrational mode. We constructed a Raman image through the intensity ratio of peak 11 for the two mutually perpendicular polarizations to investigate the twist angles of DNTT molecules in the device, as shown in Figure 4b. While the image shows large areas of uniform twist of the molecules, suggesting good transportation of the charge carriers, a variation of color localized to small areas suggests a possible degradation of charge transport in that area of the OFET device. Therefore, we found that the orientation of DNTT molecules was not perfectly uniform in the wider range of the device, where each color represents different twist angles as shown in an illustration in Figure 4c. In this way, we observed an orientation distribution of the DNTT molecules in the OFET device by using a polarization-dependent Raman spectroscopic technique. Thus, with the help of Raman spectroscopy, we can get information about the orientation of DNTT molecules in the device with a high spatial resolution of a few hundred nanometers, which would help in improving the electronic properties of the OFET device by tweaking the growth parameters.

## 4. Conclusions

In conclusion, we demonstrated a Raman measurement of DNTT molecules on a transistor device and identified the observed vibrational modes using DFT calculation. We first analyzed the Raman spectrum of DNTT molecules in which several vibrational modes were observed. One of the vibrational modes was used to investigate the molecular density distribution of DNTT in the device by means of Raman imaging and we found that the DNTT molecules did not have a uniform density distribution in the device, which may degrade the device performance. From the DFT calculation, we understood the nature of the displacements of various atoms in different vibrational modes. Our calculation indicated that the vibrational mode marked as peak 11 was dominated by the atomic displacements along a direction perpendicular to the long molecular axis of the DNTT molecules. The polarization dependence of this mode was then confirmed by using two mutually perpendicular linear polarizations within the substrate plane. From the polarization dependence of peak 11, we demonstrated the distribution of the twist angle of DNTT molecules on the surface of the OFET device. Since the variation in molecular twist affects the device performance, our study helps to optimize the fabrication process of the OFET device for better performance. As a prospect, one can also consider a tensorial Raman approach to understand the molecular orientation because the Raman tensors are expected to be influenced by the modified polarizability arising from inhomogeneity in the 3D molecular orientation. It would also be interesting to compare the tensorial approach with the present approach to understand the variation in molecular orientation. Our preliminary work on the fundamental properties of molecular vibrations will lead to new ways of analyzing various devices based on DNTT molecules, using the Raman spectroscopic technique, in order to improve their performances.

## Figures and Tables

**Table 1:** Descriptions of the vibrations in the calculated vibrational modes.

Peak	Wavenumber (cm^−1^)	Descriptions
1	1176.5	C–H bending at the end of the aromatic rings
2	1213.9	C–H bending coupled with stretching of C(14), C(17), C(15), and C(20) atoms
3	1242.5	C–H bending; hydrogen atoms in the upper part of rings bend in-phase but hydrogen atoms in the upper and the lower parts bend anti-phase, and stretching of C(14) and C(20) atoms
4	1327.8	Deformation of thiophene rings and their adjacent aromatic rings and bending of H(26), H(28), H(35), and H(36) atoms
5	1396.7	C–C anti-symmetric stretching of aromatic rings coupled with C–H bending
6	1405.8	C–C stretching coupled with C–H bending
7	1441.1	C–C symmetric stretching of aromatic rings coupled with C–H bending
8	1479.7	C–C stretching coupled with C–H bending
9	1517.0	C–C stretching coupled with C–H bending
10	1538.5	C–C stretching of the ring with a strong stretching of C(4)–C(9) atoms and C–H bending
11	1600.8	C–C symmetric stretching of the rings coupled with C–H bending
12	1635.6	Deformation of aromatic rings
13	1660.5	Deformation of the aromatic rings except for thiophene rings

## Supplementary Materials

The following are available online at http://www.mdpi.com/1996-1944/12/4/615/s1, Figure S1: Graphical representation of vibrating atoms at (a) 1242.5 cm^−1^ (peak 3), (b) 1327.8 cm^−1^ (peak 4), (c) 1441.1 cm^−1^ (peak 7), (d) 1635.6 cm^−1^ (peak 12), and (e) 1660.5 cm^−1^ (peak 13); Video S1, S2, S3, S4, S5, S6, and S7: Vibrational mode at 1242.5 cm^−1^ (peak 3), 1327.8 cm^−1^ (peak 4), 1441.1 cm^−1^ (peak 7), 1538.5 cm^−1^ (peak 10), 1600.1 cm^−1^ (peak 11), 1635.6 cm^−1^ (peak 12), and 1660.5 cm^−1^ (peak 13), respectively.
