# Supplementary material for: Raman Spectroscopic Studies of Dinaphthothienothiophene (DNTT)
Source: Materials (Basel). 2019 Feb 18;12(4):615. doi: 10.3390/ma12040615 (PMC6416593; doi:10.3390/ma12040615)
Supplement: Supplementary file 1 [file materials-12-00615-s001.zip › SI/supplementary.docx]

Supplementary

**Raman Spectroscopic Studies of Dinaphthothienothiophene (DNTT)**

**Bishwajeet Singh Bhardwaj ^1^,** **Takeshi Sugiyama ^1^, Naoko Namba ^2^,** **Takayuki Umakoshi ^1^,** **Takafumi Uemura ^2^,** **Tsuyoshi Sekitani ^2^ and Prabhat Verma ^1,^***

^1^ Department of Applied Physics, Osaka University, Suita, Osaka 565-0871, Japan; bishwajeet@ap.eng.osaka-u.ac.jp (B.S.B.), sugiyama@ap.eng.osaka-u.ac.jp (T.S.), umakoshi@ap.eng.osaka-u.ac.jp (T.U.)

^2^ The Institute of Scientific and Industrial Research, Osaka University, Mihogaoka, Ibaraki, Osaka 567-0047 Japan; nnanba@sanken.osaka-u.ac.jp (N.N.), uemura-t@sanken.osaka-u.ac.jp (T.Ue.), sekitani@sanken.osaka-u.ac.jp (T.Se.)

***** Correspondence: verma@ap.eng.osaka-u.ac.jp; Tel.: +81-6-6879-4710

Received: 19 December 2018; Accepted: 18 February 2019; Published: date

Figure S1 shows direction of the vibrational motions of each atom of a DNTT molecule at the vibrational modes, calculated by the DFT calculation. These vibrational modes were observed at 1242.5, 1327.8, 1441.1, 1635.6, and 1660.5 cm^-1^. In the vibrational mode of peak 3, all hydrogen atoms in the upper part of the aromatic rings bend in-phase, but hydrogen atoms in the upper and lower parts bend in anti-phase. In the vibrational mode of peak 4, we clearly observed deformation of thiophene rings and their adjacent aromatic ring coupled with dominating C-H in-plane bending for H(26) and H(28) atoms. There is C-C symmetric stretching of aromatic ring combined with C-H in-plane bending modes for the vibrational mode of peak 7. We observed deformation of all aromatic rings in the vibrational mode of peak 12, whereas aromatic rings at both ends of DNTT molecule mainly deforms in the vibrational mode of peak 13. These vibrational modes were also shown in the Movie files of the Supplementary Information.


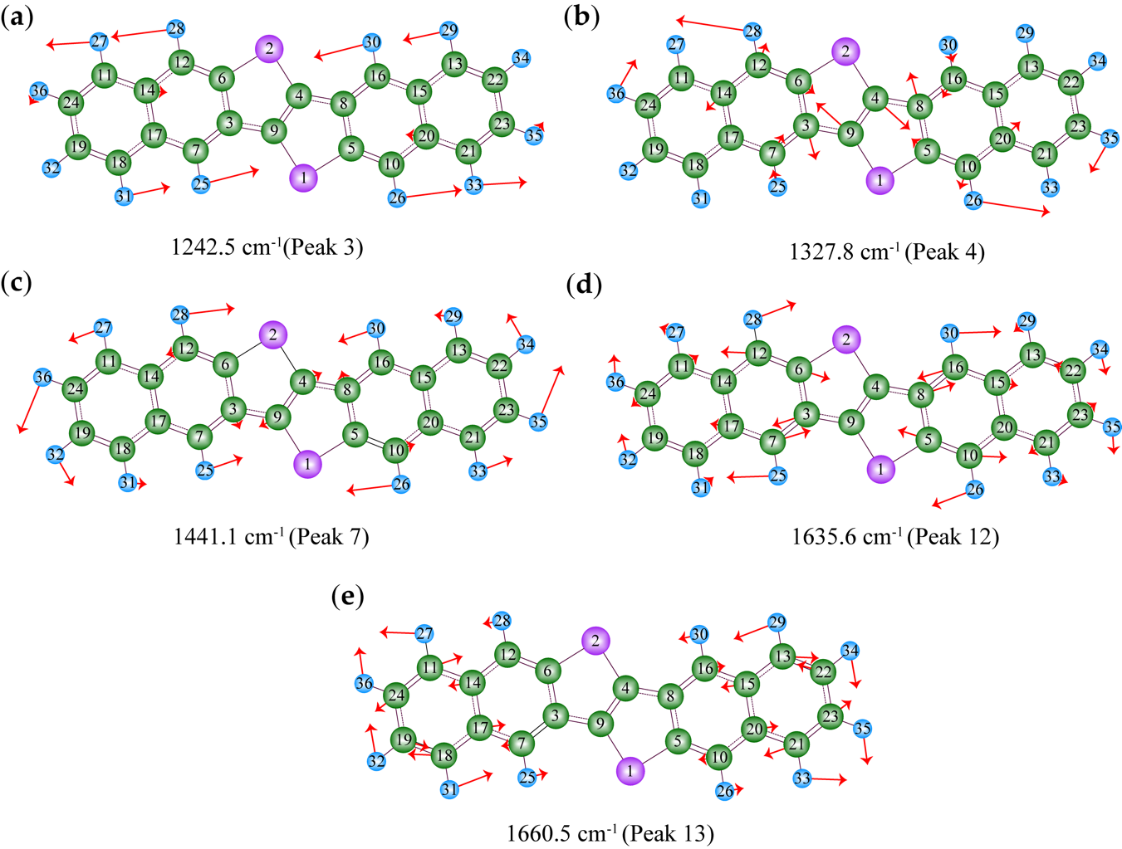


**Figure 1.** Graphical representation of vibrating atoms at (**a**) 1242.5 cm^−1^ (peak 3), (**b**) 1327.8 cm^−1^ (peak 4), (**c**) 1441.1 cm^−1^ (peak 7), (**d**) 1635.6 cm^−1^ (peak 12), and (**e**) 1660.5 cm^−1^ (peak 13).

**Video S1.** Vibrational mode at 1242.5 cm^−1^ (peak 3).

**Video S2.** Vibrational mode at 1327.8 cm^−1^ (peak 4).

**Video S3.** Vibrational mode at 1441.1 cm^−1^ (peak 7).

**Video S4.** Vibrational mode at 1538.5 cm^−1^ (peak 10).

**Video S5.** Vibrational mode at 1600.1 cm^−1^ (peak 11).

**Video S6.** Vibrational mode at 1635.6 cm^−1^ (peak 12).

**Video S7.** Vibrational mode at 1660.5 cm^−1^ (peak 13).
